# Functional Inhibition of Katanin Affects Synaptic Plasticity

**DOI:** 10.1523/JNEUROSCI.0374-23.2023

**Published:** 2023-11-22

**Authors:** Franco L. Lombino, Jürgen R. Schwarz, Yvonne Pechmann, Michaela Schweizer, Rebecca Jark, Oliver Stange, Markus Glatzel, Christine E. Gee, Torben J. Hausrat, Kira V. Gromova, Matthias Kneussel

**Affiliations:** ^1^Institute of Molecular Neurogenetics, Center for Molecular Neurobiology, ZMNH, University Medical Center Hamburg-Eppendorf, Falkenried 94, Hamburg 20251, Germany; ^2^Core Facility of Morphology and Electron Microscopy, Center for Molecular Neurobiology, ZMNH, University Medical Center Hamburg-Eppendorf, Falkenried 94, Hamburg 20251, Germany; ^3^Institute of Neuropathology, University Medical Center Hamburg-Eppendorf, Martinistraße 52, Hamburg 20246, Germany; ^4^Institute of Synaptic Physiology, Center for Molecular Neurobiology, ZMNH, University Medical Center Hamburg-Eppendorf, Hamburg 20251, Germany

**Keywords:** dendritic spine, katanin, LTP, microtubule, neuron, NMDA receptor, plasticity, PSD-95, severing, synapse

## Abstract

Dynamic microtubules critically regulate synaptic functions, but the role of microtubule severing in these processes is barely understood. Katanin is a neuronally expressed microtubule-severing complex regulating microtubule number and length in cell division or neurogenesis; however, its potential role in synaptic functions has remained unknown. Studying mice from both sexes, we found that katanin is abundant in neuronal dendrites and can be detected at individual excitatory spine synapses. Overexpression of a dominant-negative ATPase-deficient katanin subunit to functionally inhibit severing alters the growth of microtubules in dendrites, specifically at premature but not mature neuronal stages without affecting spine density. Notably, interference with katanin function prevented structural spine remodeling following single synapse glutamate uncaging and significantly affected the potentiation of AMPA-receptor-mediated excitatory currents after chemical induction of long-term potentiation. Furthermore, katanin inhibition reduced the invasion of microtubules into fully developed spines. Our data demonstrate that katanin-mediated microtubule severing regulates structural and functional plasticity at synaptic sites.

## Significance Statement

Excitatory spine synapses are rich in actin filaments that critically regulate structural and functional synaptic plasticity. In contrast, microtubules just transiently polymerize into dendritic spines. A synaptic role of dynamic microtubules is incompletely understood, and the mechanisms that regulate microtubule rearrangement at synaptic sites have remained largely unknown. Here, we show that the microtubule-severing enzyme katanin, known to keep microtubules in a dynamic state, is a component of synapses mediating functional and structural roles. Our data highlight an unnoted player of synapse function and connect microtubule severing with synaptic plasticity.

## Introduction

Dynamic microtubules (MTs) regulate axonal and dendritic transport in neurons. They control mechanisms of synaptic function, including neurotransmitter release and synaptic plasticity as well as homeostatic processes (Jaworski et al., 2009; Muhia et al., 2016; Dent, 2020; Waites et al., 2021).

MTs are polymers of α- and β-tubulin dimers that assemble in a head-to-tail fashion, resulting in filaments with plus and minus ends. At their plus ends, MTs are highly dynamic, polymerizing and depolymerizing through a stochastic process known as dynamic instability (Mitchison and Kirschner, 1984). While in most cells MTs are anchored near the centrosome, neuronal MTs can be nucleated at neurite branch points following local MT severing (McNally and Roll-Mecak, 2018). Polymeric MTs are transported by motor proteins into axons or dendrites (Wang and Brown, 2002), and MTs themselves serve as tracks for cargo transport through motor proteins (Setou et al., 2002; Hirokawa et al., 2010; Heisler et al., 2011, 2018; Guedes-Dias and Holzbaur, 2019; Dent, 2020). Notably, MT plus ends are oriented distally in axons, while anti-parallel orientations are reported in dendrites (Tas et al., 2017).

In contrast to the high abundance of MTs in neuronal dendrites (Kapitein and Hoogenraad, 2015), dendritic spines are typically rich in actin filaments (Matus, 2000; Bonhoeffer and Yuste, 2002; Zito et al., 2004). However, visualization of the microtubule + TIP protein EB3 revealed that MTs occasionally polymerize into dendritic spines at the distal dendritic regions (Hu et al., 2008; Jaworski et al., 2009) to allow KIF1A-mediated transport of synaptotagmin-IV to postsynaptic sites (McVicker et al., 2016). The MT entry into the spine protrusions lasts seconds to minutes, following NMDA-type glutamate receptor (NMDAR) activation and calcium influx (Kapitein et al., 2011; Merriam et al., 2011, 2013). Notably, chemically induced long-term potentiation (cLTP) increases these events, whereas chemically induced long-term depression (LTD) decreases them (Kapitein et al., 2011; Merriam et al., 2011, 2013).

In addition to the polymerization and depolymerization of MTs, MT severing contributes to sculpting and dynamically reorganizing MT arrays (Yu et al., 2008; McNally and Roll-Mecak, 2018). Three major AAA-ATPases mediate MT severing in neurons, known as spastin, katanin, and fidgetin (Sharp and Ross, 2012; McNally and Roll-Mecak, 2018). Of these three, we previously showed that spastin is critical for kinesin-mediated AMPA receptor transport and synaptic receptor densities, thereby regulating working and associative memory (Lopes et al., 2020).

Katanin functions as a hexameric complex of dimers assembled through 60 kDa catalytic and 80 kDa regulatory subunits (McNally et al., 2000; McNally and Roll-Mecak, 2018). In addition to its important role in cell division and adult neurogenesis (Lombino et al., 2019), mammalian katanin regulates the number and length of neuronal MTs (Sharp and Ross, 2012). It further interacts with CAMSAPs to regulate the organization and stability of MT minus ends (Jiang et al., 2018). Notably, the MT-associated protein tau protects MTs from katanin-mediated severing in axons (Qiang et al., 2006).

Here, we asked whether katanin potentially contributes to synapse function and plasticity. We found that katanin is abundant in dendrites and can be detected at actin-rich spines and glutamatergic synapses. Following dominant-negative interference with katanin-mediated severing, we observed impairments in structural spine remodeling after glutamate uncaging and reduced MT polymerization into dendritic spines. Finally, functional inhibition of katanin affected the potentiation of AMPA-receptor-mediated excitatory currents after chemical LTP induction. Our data demonstrate that katanin-mediated MT severing is essential for the regulation of synaptic plasticity.

## Materials and Methods

### Animals

Animals were maintained in the animal facility of the University Medical Center Hamburg-Eppendorf under controlled environmental conditions in accordance with the European Communities Council Directive (2010/63/EU). Harvesting of tissue and cells was performed according to the Hamburg authorities (Behörde für Justiz und Verbraucherschutz, Fachbereich Lebensmittelsicherheit und Veterinärwesen) and the animal care committee at the University Medical Center Hamburg-Eppendorf.

### Antibodies and plasmids

The following antibodies were used for immunofluorescence: P60 katanin, rabbit, 1:200 (McNally F.J.), P80 katanin, rabbit, 1:200 (Proteintech), PSD-95, guinea pig, 1:200 (Synaptic Systems), P80 katanin, mouse, 1:100 (Novus), GluA2, mouse, 1:200 (Millipore), Synaptophysin, guinea pig, 1:1,000 (Synaptic Systems), EB3, rat, 1:300 (Abcam), PSD-95, mouse, 1:300 (Invitrogen), Anti-rabbit Alexa Fluor 488, donkey (Jackson), Anti-mouse Cy3, donkey (Jackson), Anti-guinea pig Cy5, donkey (Jackson), and Anti-rat Cy5, donkey (Jackson). The following antibodies were used for electron microscopy: GFP, rabbit, 1:100 (Invitrogen) and rabbit IgG, goat, 1:1,000 (VectorLabs). The following antibodies were used for Western blotting: P60 katanin, rabbit, 1:500 (McNally F.J.), P80 katanin, rabbit, 1:500 (Proteintech), PSD-95, mouse, 1:500 (Invitrogen), GluA1, rabbit, 1:500 (Millipore), SNAP25, mouse (BD Biosciences), Beta-actin, mouse AC15 (Sigma), GAPDH, mouse, 1:1,000–2,000, 6C5 (GeneTex), Anti-mouse HRP, donkey (Jackson), Anti-rabbit HRP, donkey (Jackson), Anti-guinea pig HRP, donkey (Jackson), and Anti-rabbit HRP LC-specific (Jackson). The following plasmids were used: EGFP-p60DEID (McNally F.J.), EGFP (Takara), EB3-Tomato (Akhmanova A.), and td-Tomato (Takara).

### Cell culture and transfection

Hippocampal neurons were cultured as described in the following manner: E.16 pregnant female mice were sacrificed, and their embryos were harvested from the uterine horns. The embryos of both sexes were decapitated, and their hippocampi were dissected under the microscope. The hippocampi were subjected to 0.05% trypsin for 5 min at 37°C, followed by mechanical dissociation using a glass Pasteur pipette. Neurons were plated at different densities ∼70,000 cells per coverslip. Transfection was performed using the calcium phosphate method (Heisler et al., 2011, 2018) or using Lipofectamine 2000.

### Immunocytochemistry

Primary cultured hippocampal neurons between DIV12 and DIV17 were fixed for 4–5 min in 4% Formaldehyde/PBS and washed three times in PBS for 5 min each. Cells were permeabilized for 5 min using 0.2% Triton-X-100/PBS, followed by three washing steps of 5 min each with PBS. Blocking was performed for 1 h at room temperature with 1% BSA/PBS. Primary antibodies were incubated in 1% BSA/PBS for 1 h at room temperature. Following the three previous washing steps with PBS, secondary antibodies were incubated for 1 h in 1% BSA/PBS with or without Rhodamine/Phalloidin (1:500). Mounting was performed after three washing steps.

#### EB3 staining

The hippocampal neurons were transfected between DIV12 and DIV17, and the experiment was conducted 48 h later. The cultures were fixed with ice-cold methanol for ∼4 min, washed once with PBS at room temperature for 20 min, and post-fixed in 4% Formaldehyde/PBS for additional 4 min. Next, two washing steps with PBS were performed for a total time of 30 min, followed by blocking for 45 min at room temperature with 1% BSA/PBS. Primary antibodies were incubated overnight in a wet chamber diluted in PHEM buffer (60 mM PIPES, 25 mM HEPES, 10 mM EGTA, 2 mM MgCl_2_, pH = 6.9). After three washing steps with PBS, secondary antibodies were diluted in PHEM buffer and incubated at room temperature for 2 h. Finally, three washing steps with PBS were performed followed by mounting with Aqua Poly Mount. Imaging was achieved using an Olympus FV1000 confocal microscope. Images were quantified using Fiji (NIH, Version 2.0.0-rc-23/1.49m). When EB3 puncta were found partially inside spines or the majority of pixels of highest value were inside, the spine was considered as positive. Based on their general morphology, spines were classified into spines with heads, stubby spines, and filopodia.

#### Glycine stimulation for EB3 staining

The hippocampal cultures were transfected at DIV12 as above. Stimulation was performed for 3–5 min in Mg^2+^-free ACSF containing 50 mM Bicuculline, 1 µM TTX, and 200 µM glycine (Xu et al., 2020). Subsequently, the cells were recovered in Mg^2+^-free ACSF for 30–60 min. Immunostaining was performed as described above. Spine classification was performed based on their general morphology into spines with heads, stubby spines, and filopodia.

### Immunoelectron microscopy

Pre-embedding immunolabeling was performed as follows: Dissociated neurons were fixed with PB containing 4% paraformaldehyde and 0.1% glutaraldehyde. 2.3 M sucrose was used for cryoprotection. Penetration of immunoreagents inside the cells was obtained by two freeze–thaw cycles in liquid nitrogen. After washing in PBS, 10% horse serum (PS) containing 0.2% bovine serum albumin (BSA) was used for blocking for 15 min. Primary antibodies were incubated overnight in PBS with 1% PS and 0.2% BSA (Carrier). Cells were washed with PBS and treated with biotinylated secondary antibody diluted in Carrier solution for 90 min. Subsequently, cultures were washed and incubated with ABC (VectorLabs) 1:100 in PBS for 90 min. This was followed by washing and incubation in diaminobenzidine (DAB)-H_2_0_2_ solution (Sigma) for 10 min. Thereafter, cultures were washed three times in 0.1 M sodium cacodylate buffer (pH 7.2–7.4) followed by osmication with 1% osmium tetroxide in cacodylate buffer. Dehydration using ascending ethyl alcohol concentration steps was followed by two rinses in propylene oxide. The embedding medium was infiltrated by first immersing the sections in a 1:1 solution of propylene oxide and Epon followed by neat Epon. Finally, the samples were hardened at 60°C. The sections of the hippocampal region of 0.5 µm (semithin) were mounted onto glass slides and stained for 1 min with 1% Toluidine blue. 60 nm sections (ultrathin) were observed with a JEM-2100Plus Transmission Electron Microscope at 200 kV (JEOL). Images were acquired with the XAROSA CMOS camera (EMSIS).

### Synaptosomal fractionation

The hippocampi were isolated and homogenized at 900 rpm with 10 strokes. The homogenates were centrifugated at 4°C for 10 min at 1,000 × *g*. The supernatants were conserved, and the pellets resuspended in sucrose buffer 1 (320 mM sucrose, 1 mM NaHCO_3_, 1 mM MgCl_2_, 500 μM CaCl_2_, EDTA-free protease inhibitors) and centrifugated at 700 × *g* for 10 min at 4°C (JA-25.5 rotor). The combined supernatants were centrifugated at 13,800 × *g* for 10 min at 4°C to obtain P2 fractions, which were resuspended in sucrose buffer 2 (320 mM sucrose and 1 mM NaHCO_3_). In parallel, a sucrose gradient (0.85 M, 1 M, and 1.2 M plus 1 mM NaHCO_3_) was prepared in Ultra-Clear 14 × 95 mm, 14 ml tubes. The P2 fraction was placed on top of the gradient and centrifugated for 90 min at 82,500 × *g* and 4°C with a SW40-Ti rotor. Synaptosomal layers appeared between 1 M and 1.2 M. They were diluted fourfold in sucrose buffer 2 and centrifugated at 28,000 × *g* for 20 min at 4°C using a JA-25.50 rotor. Pellets (synaptosomes) were resuspended in 100 µl of sucrose buffer 2. For PSD fractions, synaptosomes were diluted with 2 × Triton-X-100 buffer (2% Triton-X-100, 640 mM sucrose, 24 mM Tris-HCl, pH 8.0) and shook for 15 min. The Triton-X-100-soluble fraction was separated from the Triton-X-100-insoluble fraction via centrifugation at 70,000 × *g* and 4°C for 1 h. Pellets were dried for 15 min and then resuspended in Tris-HCl pH 8.0. Samples were processed by SDS PAGE and subsequently blotted onto a PVDF membrane. Transfer was achieved in the presence of 20% methanol transfer buffer (192 mM glycine, 25 mM Tris). Blocking was performed in 5% milk in TBS-T. Antibodies were incubated either in 5% BSA in TBS-T or 5% milk in TBS-T.

### Time-lapse video microscopy, image processing

Neurons were imaged with a spinning disk microscope system (Visitron Systems GmbH), consisting of a Nikon Ti-E inverted microscope and a Yokogawa spinning disk. A chamber for temperature (37°C) and CO_2_ (5% CO_2_) control was used.

#### EB3 imaging in dendritic regions

The hippocampal cultures were co-transfected with EB3-Tomato and either GFP-p60DEID or GFP 1 day prior to imaging. Neurons were imaged at 4 days in vitro (DIV4) or DIV14, respectively. A single acquisition was obtained for the GFP channel, while for the EB3-Tomato channel, time-lapse images of 1 frame/s or 1 frame/2 s were taken. Quantification of velocity was performed with Fiji (NIH, Version 2.0.0-rc-23/1.49m) using the manual tracking plugin TrackMate. The distance of individual comets at single MTs were tracked over time to calculate average velocities. Absolute speed (no stops) was considered for analysis. Tracking was done independent of direction.

#### EB3 imaging in dendritic spines

Hippocampal neurons at DIV12 were co-transfected with EB3-Tomato and either EGFP (control) or EGFP-p60DEID. Briefly, 8 µl of Lipofectamine (Lipofectamine 2000, Invitrogen) were incubated in 50 µl of Opti-MEM. In parallel, a total of 4–8 µg of DNA were incubated in 50 µl of Opti-MEM. After 5–10 min, both reactions were mixed and administered to the transfected coverslip, after removal of ∼75% of the conditioned media. Two hours after incubation, the transfection mix was aspirated and replaced by conditioned media without washing. At DIV14, the transfected neurons were imaged in HEPES-buffered (20 mM) medium. Imaging was performed sequentially for green and red channels with an interval of 2 s between frames for a total of 300 frames. Binning of 2 was used for image acquisition. Quantification was performed using Fiji (NIH, Version 2.0.0-rc-23/1.49m). Images were processed identically throughout the experiments. The GFP channel was used to validate the expression of either EGFP-p60DEID or control. The RFP channel was used for quantification. Brightness and contrast were adjusted in order to observe neuronal morphology due to the non-comet EB3 diffusion (Kapitein et al., 2010). Individual ROIs were created and saved to mark every possible spine. Spines were classified visually in spines with heads, stubby (local deformation of the dendrite) and filopodia (very motile, long filamentous). Back in the raw image, after adjusting brightness and contrast in the same way for all the images, the ROIs of the identified spines were pasted, and each spine was analyzed individually. The presence or absence of an EB3 comet was assessed visually. EB3 invasion into spines was considered positive only if the comet fully invaded the spine and ended its trajectory in the protrusion. The length of the events and the frame in which they appeared was also considered.

### Patch-clamp recordings of EPSCs

Whole-cell patch-clamp measurements were performed on DIV12–14 hippocampal neurons. Neuron cultures were prepared from embryonic (E16) mice. Recordings were done by using borosilicate pipettes with resistances of 3.0–4.5 MΩ after filling with intracellular solution (120 mM K-gluconate, 8 mM NaCl, 2 mM MgCl_2_, 0.5 mM CaCl_2_, 5 mM EGTA, 10 mM HEPES, 14 mM phosphocreatine, 2 mM magnesium-ATP, 0.3 mM sodium-GTP, and pH adjusted to 7.3 with KOH). Patchmaster software (HEKA) in combination with an EPC-9 patch-clamp amplifier (HEKA) was used for data acquisition and pulse application. Recordings were low-pass filtered at 2.9 kHz and analyzed with Fitmaster (HEKA), Igor Pro 6.03 (WaveMetrics), Clampfit (Molecular Devices), and Excel (Microsoft). Neurons with an access resistance of <20 MΩ were evaluated. Miniature excitatory postsynaptic currents (mEPSCs) were recorded from neurons transfected either with GFP (control neurons) or with a dominant-negative mutant of katanin (GFP-p60DEID, test neurons). Current clamp experiments were done at room temperature (21–23°C) in Ringer's solution (143 mM NaCl, 5 mM 1 KCl, 0.8 mM MgCl_2_, 1 mM CaCl_2_, 10 mM HEPES, 5 mM glucose, and pH adjusted to 7.3 with NaOH). The mEPSCs were measured in the presence of TTX (0.25 μM), bicuculline (10 μM), and AP5 (20 µM), which were added to the Ringer's solution. All substances were purchased from Sigma Aldrich. Chemical LTP was induced with the following protocol: Neurons were preconditioned with the NMDA antagonist AP5 (50 µM) for 24 h before patch-clamp experiments. After obtaining the whole-cell configuration and recording of the resting potential and action potentials, the mEPSCs were recorded for 5 min at resting conditions. Thereafter, neurons were stimulated with a series of 10 depolarizing pulses of 70 mV amplitude and 100 ms duration with intervals of 50 ms. During the electrophysiological stimulation, the Ringer's solution was changed to a Mg^2+^-free solution containing 200 µM glycine and 3 mM CaCl_2_ (Lu et al., 2001). Only experiments that were stable for 30 min after stimulation were evaluated. The mEPSC amplitudes and inter-event intervals (IEI) were determined by using Clampfit (Molecular Devices) and summarized in bins of 5 min.

### Two-photon glutamate uncaging

E16 embryos were used for the preparation of primary hippocampal neurons in culture. Cells were transfected with either GFP-p60DEID/mCherry or GFP/mCherry (control) at DIV 12 using Lipofectamine (Lipofectamine 2000, Invitrogen) or a calcium phosphate protocol. After 24–48 h of expression, two-photon glutamate uncaging was performed on an Olympus FV1000 microscope controlled by the FluoView software. Excitation was achieved by a pulsed Ti:sapphire laser (Mai Tai, Spectra Physics) tuned to 880 or 950 nm. Four Z-stack images were collected with an Olympus XLPlanN 25X MP, 1.05 NA water dipping objective and a 495–540 nm emission filter. Experiments were conducted in Mg^2+^-free Ringer solution (125 mM NaCl, 2.5 mM KCl, 2 mM CaCl_2_, 33 mM D-glucose, 25 mM HEPES, pH 7.3) in the presence of 2.5 mM MNI-caged-L-glutamate (Tocris) and 0.001 mM TTX (Tocris). Two to four dendritic spines were targeted for potentiation in each neuron. After imaging of baseline over approximately 10 min, glutamate was uncaged by setting the laser to 720 nm with 60 pulses, 1 ms at 1 Hz. Following uncaging, the spine volume was monitored for 20 min.

### Statistical analysis

Data exploration, statistical analysis and graphical representation were performed with GraphPad Prism 9.0.1.

#### EB3 time-lapse imaging in DIV4 and DIV14 neurons

Data were explored using descriptive statistics and normal distribution. The Anderson–Darling test was used to assess normality of the data. The two-tailed Mann–Whitney *U* test was used to assess significance.

#### Endogenous katanin and EB3 in dendritic spines

The Anderson–Darling test was used to assess for a normal distribution. The two-tailed Mann–Whitney *U* test was applied between conditions “no p60-no EB3” versus “yes p60–yes EB3”.

#### Endogenous EB3 in dendritic spines upon GFP-p60DEID overexpression and EB3-Tomato time-lapse in dendritic spines

A parametric *t* test was performed following assessment of a normal distribution. If data showed a not normal distribution, a non-parametric Mann–Whitney *U* test was applied.

#### Patch-clamp recordings of EPSCs

Outliers were analyzed by GraphPad. For peak amplitudes, the percentage was calculated and normalized prior to the stimulation time point. Significant interactions were observed between the time and condition using two-way ANOVA. For IEI, fold change was calculated with respect prior to the stimulation time point (set to 1).

#### Two-photon glutamate uncaging

Spine areas were marked by drawing freehand regions around the spine perimeter. To assess changes in spine size, the average of the area from individual spines during the baseline was set as 100%. Normalization was performed for each individual time point. Outliers were removed with SPSS. GraphPad was used to compute two-way ANOVA. Significance was assessed by multiple comparisons with the uncorrected Fisher's LSD test.

## Results

### Katanin inhibition affects microtubule growth in dendrites of premature but not mature neurons

**Figure 1.**
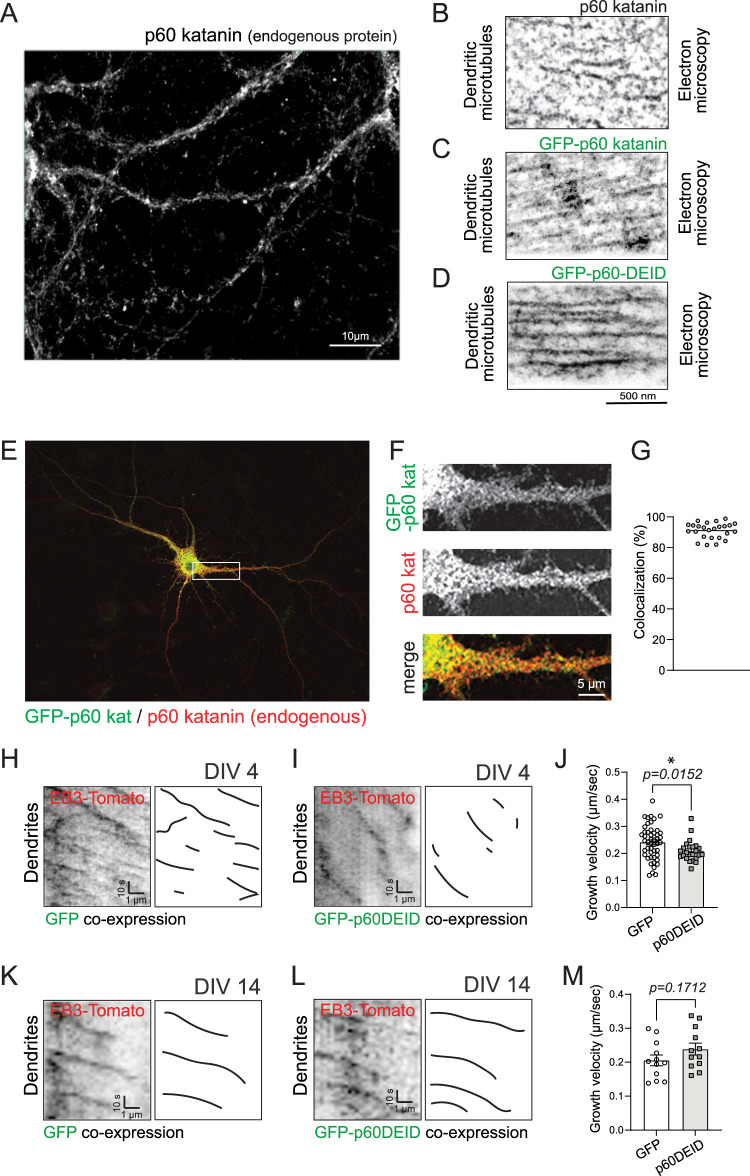
Katanin inhibition affects MT growth in dendrites of premature but not mature neurons. ***A***, Immunostaining of endogenous p60 katanin in dendrites of cultured hippocampal neurons at DIV 13–17. *n* = 3 experiments. ***B***, Anti-p60 katanin immunoelectron microscopy with diaminobenzidine (DAB) showing MTs from hippocampal neurons. ***C,D***, Anti-GFP immunoelectron microscopy with diaminobenzidine (DAB) showing MTs from hippocampal neurons transfected with either GFP-p60 katanin (***C***) or GFP-p60DEID (***D***). ***E***, Overview image of GFP-p60 katanin (green) and endogenous p60 katanin (red). ***F***, Magnifications of boxed region in ***E***. ***G****,* Quantification of GFP-p60 katanin/p60 (endogenous) katanin colocalization levels. Mean ± S.E.M. = 91.16 ± 1.006%. Three independent experiments. ***H,I***, EB3-Tomato time-lapse imaging in dendrites of DIV4 hippocampal neurons. Right: diagrams of EB3 tracks for GFP in ***H*** and for GFP-p60DEID in ***I***. Neurons express EB3-Tomato (red) with either GFP or GFP-p60DEID (green). The green channel is not shown. ***J***, Quantification of EB3 growth velocity (µm/s). GFP: 0.24 ± 0.01, GFP-p60DEID: 0.21 ± 0.01. Mann–Whitney *U* test *p* = 0.0152. GFP: *n* = 48 comets; GFP-p60DEID: *n* = 24 comets, three independent experiments. ***K,L***, EB3-Tomato time-lapse imaging in dendrites of DIV14 hippocampal neurons. Right: diagrams of EB3 tracks for GFP in ***K*** and for GFP-p60DEID in ***L***. Neurons express EB3-Tomato (red) with either GFP or GFP-p60DEID (green). The green channel is not shown. ***M***, Quantification of ***K*** and ***L*** shows comparable growth velocity among conditions. GFP: 0.2054 + 0.01584 μm/s, GFP-p60DEID: 0.2387 + 0.01736 μm/s, Unpaired *t* test, two-tailed, *p* = 0.1712, (GFP: *n* = 12 ROIs, 503 comets); (GFP-p60DEID: *n* = 12 ROIs, 340 comets), four independent experiments.

Katanin severs MTs in the regulation of a dynamic MT cytoskeleton (Sharp and Ross, 2012; McNally and Roll-Mecak, 2018). We found that endogenous katanin is abundantly expressed in neuronal dendrites (Fig. 1*A*) and labels MTs (Fig. 1*B*). Therefore, we asked whether it contributes to the dynamics of dendritic MTs. To interfere with katanin-mediated MT severing, we overexpressed a validated dominant-negative katanin construct of the catalytic p60 subunit (GFP-katanin p60DEID) that is known to prevent ATP hydrolysis and consequently MT-severing activity (DEID ≙ dead) (McNally et al., 2000). Control experiments applying DAB immunoelectron microscopy confirmed that fluorescent GFP-fusion proteins of wild-type p60 katanin and p60DEID decorate MTs in neuronal dendrites (Fig. 1*C,D*). Besides, GFP-p60 katanin colocalizes with the endogenous protein using immunocytochemistry (Fig. 1*E–G*). Interestingly, live-cell imaging with the fluorescent MT + TIP fusion protein EB3-Tomato revealed that katanin inhibition significantly reduces MT growth velocities in premature stage IV neurons at DIV4 (Dotti et al., 1988; Baj et al., 2014), but does not affect MT growth in mature stage VI neurons at DIV14 (Fig. 1*H–M*). These data are consistent with a functional role of katanin during neuronal development (Yu et al., 2005). Thus, katanin is critical during dendritogenesis, but seems to be less important for the regulation of MT growth in dendrites of mature neurons.

### Katanin is located at glutamatergic spine synapses

**Figure 2.**
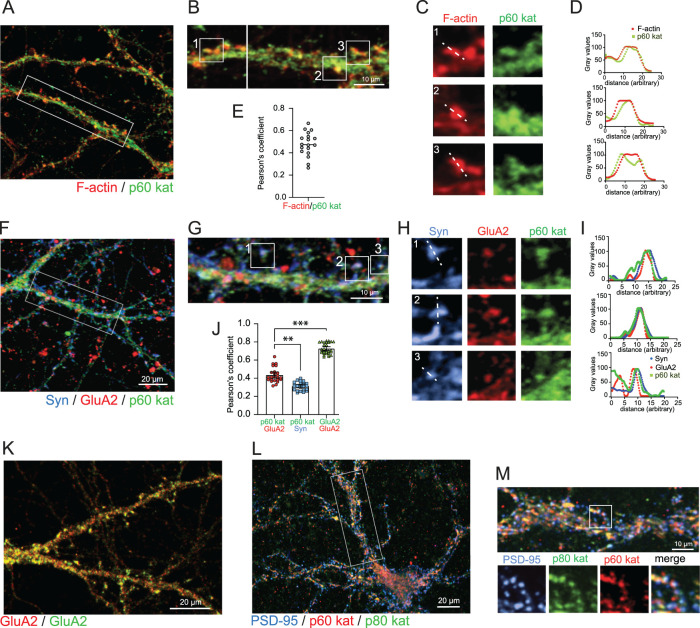
p60 katanin is located at glutamatergic spine synapses. ***A,B***, Immunostaining of endogenous p60 katanin (green), Rhodamine phalloidin labeling of F-actin (red), DIV13–17 neurons, three independent experiments. ***C***, Magnifications of spines from boxed regions in ***B***. ***D***, Fluorescence intensity profiles of line scans in ***C***. ***E***, Pearson's correlation coefficient indicating colocalization between p60 katanin and F-actin. Mean ± S.E.M = 0.4797 ± 0.02536, *n* = 18 images. ***F,G***, Triple-immunostaining, endogenous p60 katanin (green), AMPAR subunit GluA2 (red), presynaptic marker synaptophysin (Syn, blue), DIV16–17 neurons, three independent experiments. ***H***, Magnification of boxed regions in ***G***. ***I***, Fluorescence intensity profiles of line scans in ***H***. ***J***, Pearson's correlation coefficient between p60 and GluA2, p60 and Syn, or GluA2 (red) and GluA2 (green). p60/GluA2: mean ± S.E.M = 0.4362 ± 0.01646, *n* = 24 ROIs; p60/Syn: mean ± S.E.M = 0.3144 ± 0.009337, *n* = 24 ROIs; GluA2/GluA2: mean ± S.E.M = 0.8273 ± 0.01246, *n* = 30 ROIs. Kruskal–Wallis test: p60/GluA2 versus p60/Syn *p* = 0.0025; Kruskal–Wallis test: p60/GluA2 versus GluA2/GluA2 *p* < 0.0001, three independent experiments. ***K***, Immunostaining of AMPA receptor GluA2 subunits with two fluorophores (red and green) as a positive control for the Pearson's correlation coefficient shown in Figure 2*J*. ***L,M***, Triple immunostaining of p60 katanin (red), p80 katanin (green) and PSD-95 (blue). The boxed region magnified below depicts triple colocalized puncta.

**Figure 3.**
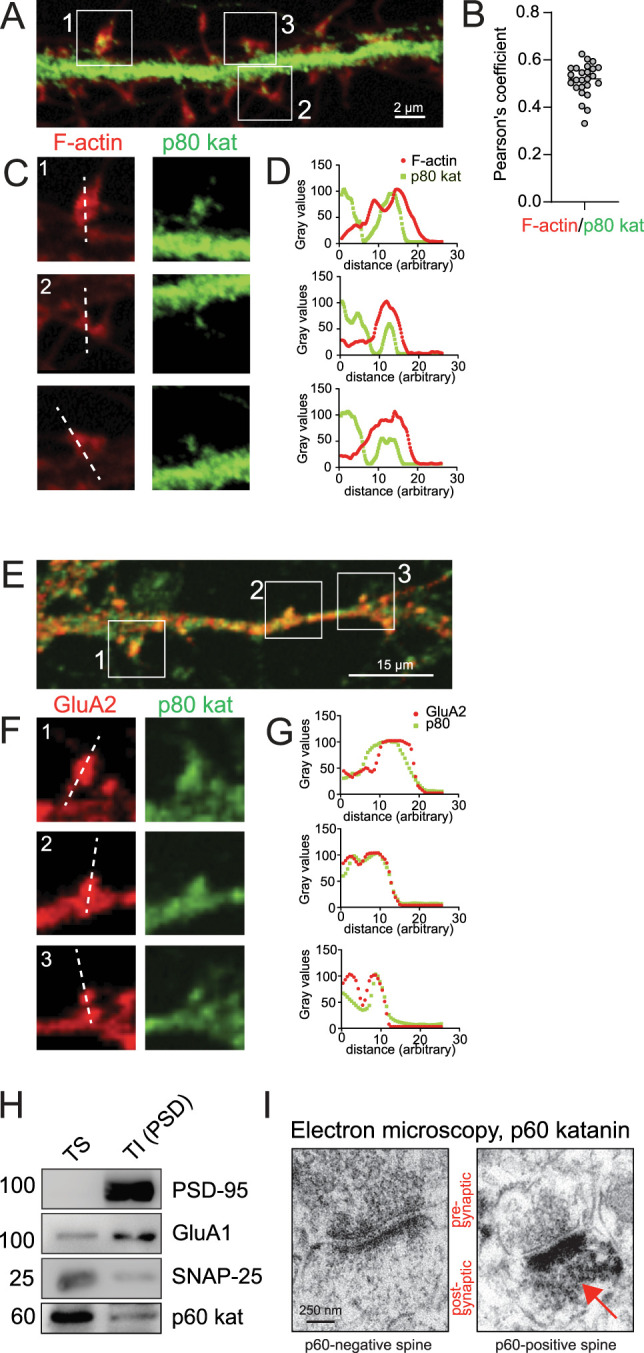
p60 and p80 katanin are located at excitatory spine synapses. ***A***, Immunostaining of endogenous katanin p80 (green) and Rhodamine phalloidin labeling of F-actin (red), DIV13 neurons, three independent experiments. ***B***, Pearson's correlation coefficient indicating colocalization of p80 katanin and F-actin. Mean ± S.E.M = 0.5149 ± 0.01379, *n* = 25 ROIs. ***C***, Magnifications of spines from boxed regions in ***A***. ***D***, Fluorescence intensity profiles of line scans in ***C***. ***E***, Co-immunostaining of endogenous p80 katanin (green) and AMPAR subunit GluA2 (red), DIV13–16 neurons, three independent experiments. ***F***, Magnification of boxed regions in ***E***. ***G***, Fluorescence intensity profiles of line scans in ***F***. ***H***, Western blot analysis of p60 katanin in Triton-X-100-soluble (TS) and Triton-X-100-insoluble (TI) synaptosomal fraction. The TI fraction is also known as PSD fraction, which is enriched for postsynaptic markers (PSD-95, GluA1), but contains very little presynaptic marker (SNAP-25). *n* = 3 experiments. ***I***, Anti-p60 katanin immunoelectron microscopy with diaminobenzidine (DAB) showing a katanin-negative (left) next to a katanin-positive (right, red arrow) spine synapse from hippocampal neurons.

Co-immunostaining of p60 katanin and F-actin revealed that the catalytic katanin subunit is detectable in 24.94 ± 6.94% of all spine protrusions (Fig. 2*A–D*) with a Pearson's correlation coefficient of 0.48 (Fig. 2*E*). In order to assess whether these protrusions represent glutamatergic synapses, we performed triple immunostaining against the presynaptic marker synaptophysin (Syn) and the postsynaptic AMPA receptor subunit GluA2. The p60 katanin colocalized with both markers at the tips of spines (Fig. 2*F–I*) with Pearson's correlation coefficients of 0.59 (Fig. 2*J,K*). We further performed triple immunostaining of p60 katanin, p80 katanin, and the synapse marker PSD-95 to prove that both the catalytic and the regulatory katanin subunit colocalize at synaptic sites (Fig. 2*L,M*). The p80 katanin likewise colocalized with F-actin and the AMPA receptor subunit GluA2 (Fig. 3*A–G*) within dendritic spines.

In addition, we prepared synaptosomal fractions to confirm that katanin is present in synapses. Consistent with the imaging data, p60 katanin was found at triton-insoluble (TI) postsynaptic densities (PSDs) that contained PSD-95 and the AMPA receptor subunit GluA1 (Fig. 3*H*, right). Likewise, p60 katanin was present in triton-soluble (TS) fractions, containing soluble pre- and postsynaptic proteins (Fig. 3*H*, left). Finally, electron microscopy revealed that katanin is mainly located at postsynaptic sites of individual spine synapses (Fig. 3*I*, right).

### Katanin inhibition does not affect spine density but interferes with glutamate-induced structural spine remodeling

**Figure 4.**
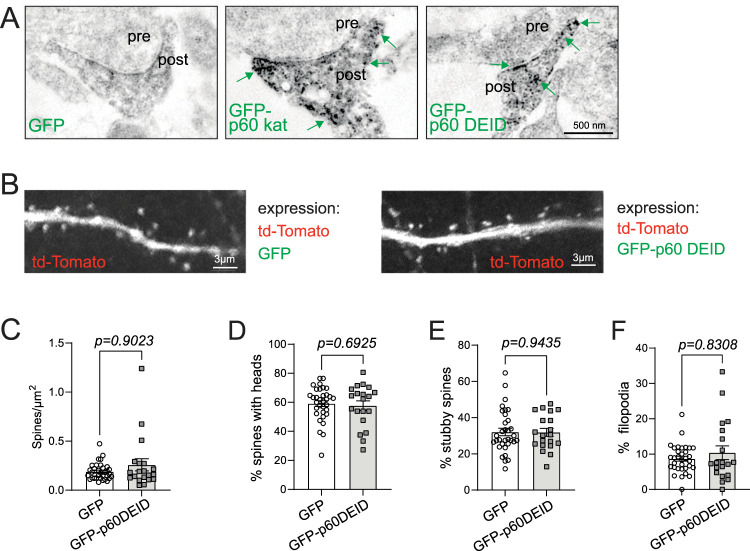
Analysis of GFP-p60DEID expression regarding spine density and structure. ***A***, DAB immunoelectron microscopy of dendritic spines of hippocampal cultures transfected either with GFP, GFP-p60 katanin, or GFP-p60DEID katanin. ***B***, Hippocampal neurons at DIV 12–17 transfected with td-Tomato as volume marker to visualize dendritic spines and either GFP or GFP-p60DEID. Please note that the red channel (td-Tomato) is shown, whereas the green channel is not shown. ***C***, Quantification of spine density (number of spines/µm^2^). GFP: 0.19 ± 0.01%, GFP-p60DEID: 0.26 ± 0.06%. Mann–Whitney *U* test *p* < 0.9023 (GFP: *n* = 33 cells, GFP-p60DEID *n* = 20 cells), three independent experiments. ***D***, Quantification of % spines with heads. GFP: 59.16 ± 2.01%, GFP-p60DEID: 57.72 ± 3.23%, unpaired two-tailed *t* test *p* = 0.6925 (GFP: *n* = 33 cells, GFP-p60DEID: *n* = 20 cells), three independent experiments. ***E***, Quantification of percentage stubby spines. GFP: 32.10 ± 2.08%, GFP-p60DEID: 31.88 ± 2.19%, unpaired two-tailed *t* test *p* = 0.9435 (GFP: *n* = 33 cells, GFP-p60DEID: *n* = 20 cells), three independent experiments. ***F***, Quantification of percentage filopodia. GFP: 8.70 ± 0.70%, GFP-p60DEID: 10.40 ± 1.95%, Mann–Whitney *U* test *p* = 0.8308 (GFP: *n* = 33 cells, GFP-p60DEID: *n* = 20 cells), three independent experiments.

Prior to inhibit katanin function with the dominant-negative p60DEID subunit (McNally et al., 2000), we aimed to check whether the mutant protein enters spines and might in general be detectable at synaptic sites. Control experiments using immunoelectron microscopy with DAB confirmed the postsynaptic localization of wild-type GFP-p60 katanin at ultrastructural resolution (Fig. 4*A*, middle, green arrows) and further revealed that the dominant-negative katanin mutant is detectable at neuronal synapses (Fig. 4*A*, right, green arrows). These data demonstrate that katanin associates with postsynaptic sites irrespective of its severing activity.

**Figure 5.**
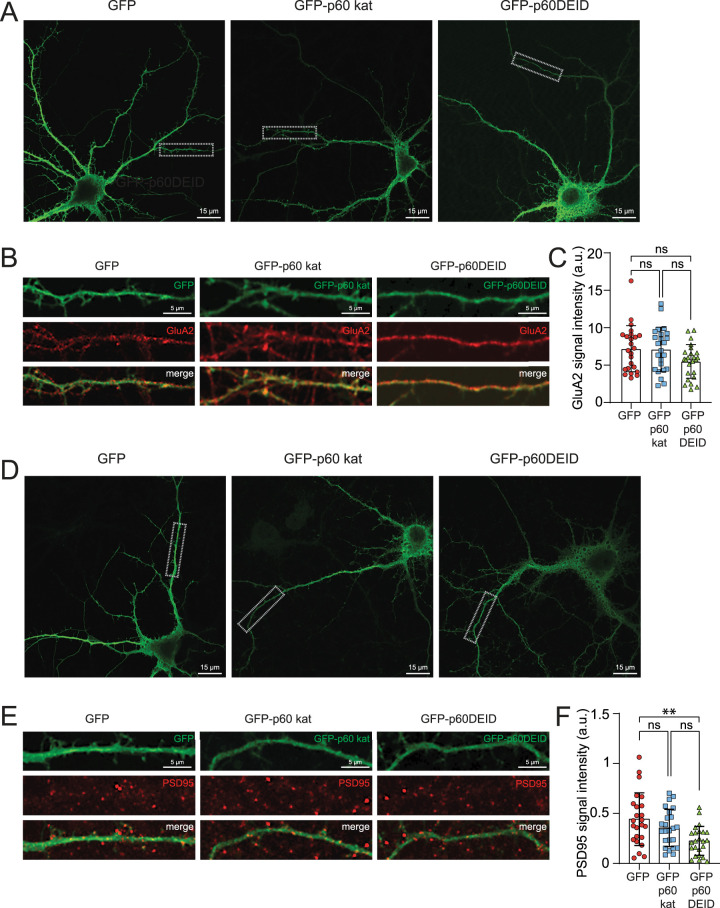
Analysis of GluA2 and PSD-95 signal intensities following overexpression of GFP-p60DEID. ***A,B***, Hippocampal neurons depicting endogenous GluA2 (red), expressing either GFP (left), GFP-p60 katanin (middle) or GFP-p60DEID katanin (right), respectively. Scale bars: 15 µm (upper overview images) and 5 µm (magnifications). ***C***, Quantification of ***B***, the signal intensity of GluA2 in 30 µm sections of secondary dendrites of hippocampal cultures transfected either with GFP, GFP-p60 katanin, or GFP-p60DEID katanin. GFP: mean ± S.E.M = 7.199 ± 0.628, *n* = 24 ROIs; GFP-p60 katanin: mean ± S.E.M = 7.109 ± 0.604, *n* = 24 ROIs; GFP-p60DEID: mean ± S.E.M = 5.490 ± 0.461, *n* = 24 ROIs, three independent experiments. Kruskal–Wallis test followed by Dunn's multiple comparison test: GFP versus GFP-p60 katanin *p* > 0.9999; GFP versus GFP-p60DEID *p* = 0.1604; GFP-p60 katanin versus GFP-p60DEID *p* = 0.1579. ***D,E***, Hippocampal neurons depicting endogenous PSD-95 (red), expressing either GFP (left), GFP-p60 katanin (middle), or GFP-p60DEID katanin (right), respectively. Scale bars: 15 µm (upper overview images) and 5 µm (magnifications). ***F***, Quantification of ***E***, the signal intensity of PSD-95 in 30 µm sections of secondary dendrites of hippocampal cultures transfected either with GFP, GFP-p60 katanin, or GFP-p60DEID katanin. GFP: mean ± S.E.M = 0.443 ± 0.054, *n* = 24 ROIs; GFP-p60 kat: mean ± S.E.M = 0.355 ± 0.038, *n* = 24 ROIs; GFP-p60DEID: mean ± S.E.M = 0.227 ± 0.029, *n* = 24 ROIs, three independent experiments. Ordinary one-way ANOVA test followed by Sidak's multiple comparison test: GFP versus GFP-p60 katanin *p* = 0.3603; GFP versus GFP-p60DEID *p* = 0.0014; GFP-p60 katanin versus GFP-p60DEID *p* = 0.0968.

Analysis of spine densities in the presence or absence of katanin GFP-p60DEID revealed equal spine numbers/µm^2^ under both conditions (Fig. 4*B,C*). We further quantified spines with heads versus stubby spines and filopodia (Fig. 4*D–F*), but did not detect changes in the density of any spine population, suggesting that katanin-mediated severing does per se not regulate spine formation or retrieval. Finally, the expression of GFP-p60 katanin or GFP-p60DEID did not alter GluA2-type AMPA receptor levels (Fig. 5*A–C*); however, katanin inhibition through GFP-p60DEID reduced PSD-95 levels (Fig. 5*D–F*). We therefore wondered whether spine structure might be sensitive to functional katanin.

**Figure 6.**
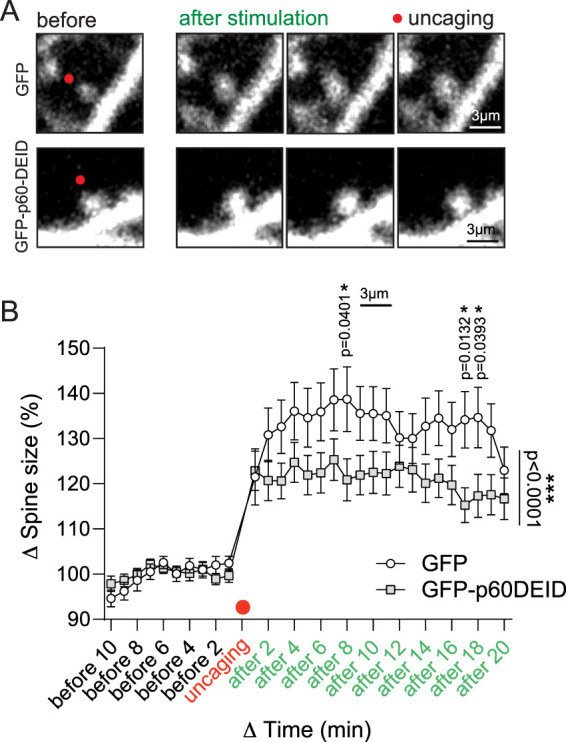
Inhibition of katanin function interferes with glutamate-induced structural spine remodeling. ***A***, Glutamate uncaging at single synapses using cultured hippocampal neurons that express either GFP or GFP-p60DEID. Left: before stimulation (black), right: after stimulation (green). Red dot: time point of uncaging stimulus. ***B***, Quantification shows a significant difference in spine size (Δ spine size) between GFP and GFP-p60DEID after stimulation. two-way ANOVA test, significant interaction between time and condition, *p* < 0.0001, multiple comparison with Fisher's LSD test shows significant effects “after 8 min” *p* = 0.0401; “after 17 min” *p* = 0.0132; “after 18 min” *p* = 0.0393 (GFP, *n* = 39 spines from 14 cells; GFP-p60DEID *n* = 45 spines from 16 cells), 2 min bins. Before uncaging 10–0 min (black axis labels), after uncaging 0–20 min (green axis labels), five independent experiments. Individual *p*-values of ***B***: before 10: *p* = 0.2037, before 9: *p* = 0.3285, before 8: *p* = 0.643, before 7: *p* = 0.4352, before 6: *p* = 0.4912, before 5: *p* = 0.8337, before 4: *p* = 0.4603, before 3: *p* = 0.9628, before 2: *p* = 0.1799, before 1: *p* = 0.2105, after 1: *p* = 0.8589, after 2: *p* = 0.1784, after 3: *p* = 0.0958, after 4: *p* = 0.1491, after 5: *p* = 0.1095, after 6: *p* = 0.0886, after 7: *p* = 0.1137, after 8: *p* = 0.0401, after 9: *p* = 0.0694, after 10: *p* = 0.0904, after 11: *p* = 0.1002, after 12: *p* = 0.4043, after 13: *p* = 0.3584, after 14: *p* = 0.0993, after 15: *p* = 0.0769, after 16: *p* = 0.1022, after 17: *p* = 0.0132, after 18: *p* = 0.0393, after 19: *p* = 0.0591, after 20: *p* = 0.3665.

The structural remodeling of dendritic spines is driven by their dynamic actin cytoskeleton (Alvarez and Sabatini, 2007; Cingolani and Goda, 2008); however, to which extent katanin-mediated functions and/or MTs contribute to spine structure is incompletely understood. Glutamate can be sufficient to trigger dendritic spine growth from dendrite shafts a process that requires opening of NMDARs and activation of cAMP-dependent protein kinase (PKA) (Kwon and Sabatini, 2011). To further investigate whether neuronal activity-induced spine remodeling (Alvarez and Sabatini, 2007; Cingolani and Goda, 2008) requires functional katanin, we applied two-photon glutamate uncaging at single spines of hippocampal neurons that expressed td-Tomato as a volume marker. Following glutamate uncaging onto single spine synapses, we observed rapid spine growth (<2 min) that persisted at least 20 min (Fig. 6*A,B*, open circles). In neurons overexpressing the dominant-negative katanin mutant GFP-p60DEID (McNally et al., 2000), the glutamate-uncaging-induced growth of spine protrusions was strongly inhibited (Fig. 6*A,B*, gray squares), suggesting that katanin-mediated functions critically contribute to the activity-dependent remodeling of spines.

### Dominant-negative inhibition of katanin function reduces microtubule polymerization into dendritic spines

A previous study reported that activity-dependent actin remodeling at the base of dendritic spines promotes MT entry (Schatzle et al., 2018). Since the base of spines represents a highly interconnected actin–microtubule interface (Korobova and Svitkina, 2010), we therefore asked whether katanin might affect MT polymerization into spines.

**Figure 7.**
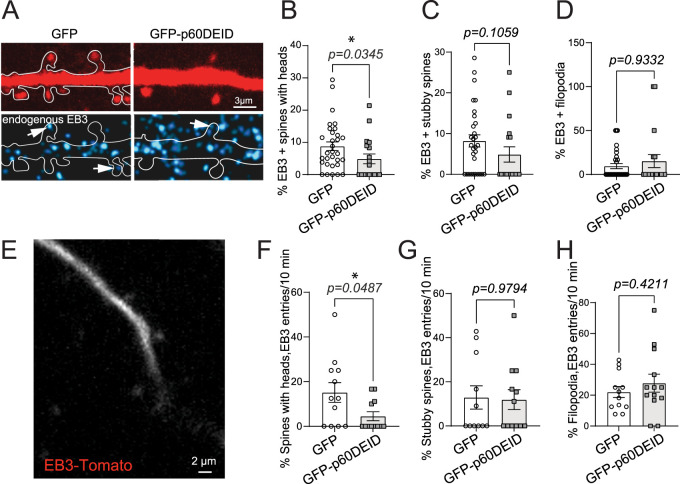
Functional inhibition of katanin reduces microtubule polymerization into dendritic spines. ***A***, Immunostaining of endogenous EB3 (blue) following expression of td-Tomato (volume marker, red) together with either GFP or GFP-p60DEID (green channel not shown), DIV14 neurons. ***B***, Quantification of % of EB3-positive spines with heads. GFP: 8.81 ± 1.35%, GFP-p60DEID: 4.90 ± 1.53%, Mann–Whitney *U* test *p* = 0.0345 (GFP: *n* = 33 cells, 979 spines; GFP-p60DEID: *n* = 20 cells, 402 spines). ***C***, Quantification of percentage of EB3-positive stubby spines. GFP: 8.18 ± 1.50%, GFP-p60DEID: 4.86 ± 1.87%, Mann–Whitney *U* test, *p* = 0.1059 (GFP: *n* = 33 cells, 505 spines; GFP-p60DEID: *n* = 20 cells, 187 spines). ***D***, Quantification of percentage of EB3-positive filopodia. GFP: 9.52 ± 2.90%, GFP-p60DEID: 15.26 ± 7.43%, Mann–Whitney *U* test, *p* = 0.9332 (GFP: *n* = 33 cells, 137 spines; GFP-p60DEID: *n* = 20 cells, 59 spines), three independent experiments. ***E***, Time-lapse microscopy to investigate EB3 invasion into spines (compare with movie 7-1). Please note that the signal intensity of this example is shown with increased brightness to visualize the transient invasion and removal of the red EB3 signal (boxed region). ***F–H***, Quantification of time-lapse video microscopy using EB3-Tomato. Please note the imaging period was 10 min, four independent experiments. ***F***, Percentage of spines with heads invaded by EB3-Tomato over 10 min: GFP: 15.16 ± 4.43%, GFP-p60DEID: 4.54 ± 2.01%, Mann–Whitney *U* test, *p* = 0.0487 (GFP: *n* = 12; GFP-p60DEID: *n* = 13 cells). ***G***, Percentage of stubby spines invaded by EB3-Tomato over 10 min: GFP: 12.90 ± 5.29%, GFP-p60DEID: 11.93 ± 4.51%, Mann–Whitney *U* test, *p* = 0.9794 (GFP: *n* = 12; GFP-p60DEID: *n* = 13 cells). ***H***, Percentage of filopodia invaded by EB3-Tomato over 10 min: GFP: 22.07 ± 3.58%, GFP-p60DEID: 27.78 ± 5.82%, Unpaired two-tailed *t* test *p* = 0.4211 (GFP: *n* = 12; GFP-p60DEID: *n* = 13 cells), four independent experiments.

To this end, we first performed immunostaining with an EB3-specific antibody in hippocampal neurons expressing a volume marker to visualize spine protrusions (Fig. 7*A*). Notably, functional inhibition of katanin-mediated microtubule severing (McNally et al., 2000) specifically reduced the percentage of EB3-positive (EB+) signals in spines with a head (Fig. 7*B*), but not in stubby spines or filopodia (Fig. 7*C,D*).

**Movie 7-1:** EB3-Tomato-positive comet representing microtubule polymerization into a dendritic spine. The spine is highlighted by an arrow in Figure 5E. Frame rate: 7 frame/s.

Using EB3-Tomato, we further visualized MT polymerization into dendritic spines in live-cell imaging experiments (Fig. 7*E* and Extended Data Movie 7-1). Whereas former studies applied image acquisition times of 3 min (Hu et al., 2008; Jaworski et al., 2009), we imaged over 10 min periods. Consistent with the EB3 immunostaining data, live imaging confirmed a significant reduction of MT entries into spines with heads when katanin function was inhibited by GFP-p60DEID (Fig. 7*F*). In contrast, MT polymerization into stubby spines or filopodia (Fig. 7*G,H*) remained unaltered in GFP-p60DEID-expressing neurons.

These data suggest that functional inhibition of katanin can in general affect MT polymerization into spines with heads, a subtype that is typically characterized by abundant amounts of glutamate receptors and high glutamate sensitivity (Matsuzaki et al., 2001) (compare with Fig. 6).

### Katanin regulates neuronal function and plasticity at potentiated synapses

**Figure 8.**
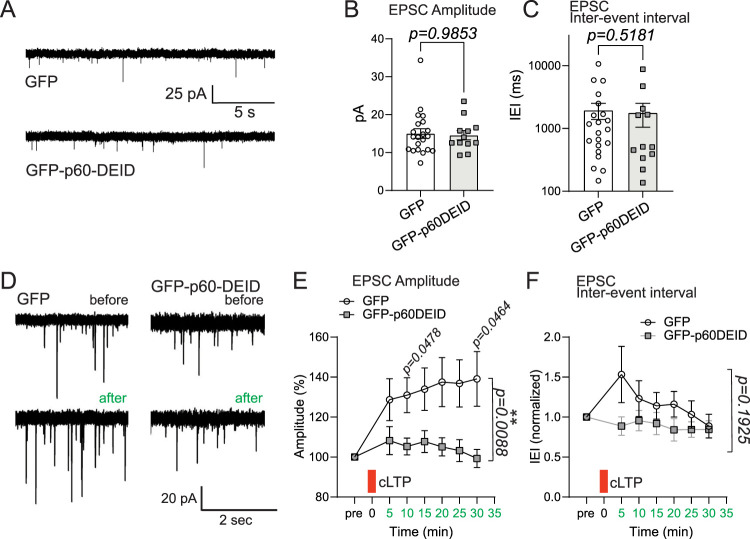
Functional inhibition of katanin alters synaptic transmission. ***A***, Katanin GFP-p60DEID overexpression in primary hippocampal neurons does not alter mEPSCs under basal conditions, GFP: *n* = 21 neurons, GFP-p60DEID: *n* = 12 neurons, three independent experiments. ***B***, Mean amplitudes (GFP: 15.03 ± 1.26 pA; GFP-p60DEID: 14.57 ± 1.21 pA, Mann–Whitney *U* test *p* = 0.9853. ***C***, Medians of inter-event intervals (IEI). GFP: 1,952 ± 565.2 ms; GFP-p60DEID: 1,780 ± 736.3 ms, Mann–Whitney *U* test *p* = 0.5181. ***D***, Katanin p60DEID overexpression in primary hippocampal neurons suppresses synaptic potentiation (cLTP). three independent experiments. ***E***, EPSC amplitudes (pA). Stimulation increases the amplitudes of control transfected cells. GFP control, directly after cLTP induction: 128%; GFP control, 30 min after cLTP induction: 139%; GFP-p60DEID, directly after cLTP induction: 108%; GFP-p60DEID, 30 min after cLTP induction: 99%. 2-way ANOVA: time × condition interaction *p* = 0.0088. Individual data points *t* tests: 10 min: *p* = 0.0478; 30 min: *p* = 0.0464. 5 min bins. ***F***, Inter-event intervals (IEIs). two-way ANOVA: time × condition no interaction *p* = 0.1925. Bins: 5 min. Red bar: time point of cLTP stimulus.

Dendritic spine synapses are fundamental signaling units that mediate communication between individual neurons within neuronal circuits, and process information through long-term modification of their strength and structure. We therefore asked whether interference with MT severing (McNally et al., 2000) might alter synaptic transmission in hippocampal neurons at DIV12–14. An analysis of AMPAR-mediated miniature excitatory postsynaptic currents (mEPSCs) under basal conditions revealed comparable mEPSC amplitudes in the presence (GFP) or absence (GFP-p60DEID) of functional katanin (Fig. 8*A,B*). Likewise, the inter-event intervals were equal under both conditions (Fig. 8*A,C*), indicating that basal synaptic transmission is independent of functional katanin.

Katanin function, however, is required for plasticity of mEPSCs. Activation of NMDARs through the removal of Mg^2+^ and the application of glycine (cLTP) (Lu et al., 2001) significantly increased mEPSC amplitudes, but not their frequency (inter-event intervals) (Fig. 8*D–F*, open circles). Notably, functional inhibition of katanin's ATPase activity (GFP-p60DEID overexpression) completely prevented cLTP (Fig. 8*D–F*, gray squares). Thus, katanin-mediated microtubule severing is a prerequisite for synaptic potentiation in primary neurons.

In summary, our data identify the MT-severing complex katanin at neuronal synapses and show that inhibition of katanin function interferes with activity-dependent structural spine remodeling and cLTP.

## Discussion

Here, we show that the MT-severing factor katanin is located at neuronal synapses and mediates critical roles in regulating synapse structure and function, specifically under activity-dependent conditions. Within the hexameric katanin protein complex, the catalytic p60 subunit can be replaced by two alternative p60-like subunits (McNally and Roll-Mecak, 2018; Shin et al., 2019). Since genetic triple knockouts of p60 and its homologues are unavailable, we alternatively applied functional inhibition of katanin's ATPase activity with a dominant-negative approach that has been shown to inhibit MT severing (McNally et al., 2000).

Using cultured primary neurons, we first analyzed MT dynamics in dendrites. At stage DIV4, when dendrite outgrowth occurs, but neurons do not yet have dendritic spines (Dotti et al., 1988), katanin inhibition altered MT growth velocities (Fig. 1), suggesting that katanin-mediated functions might contribute to dendritogenesis at early developmental stages. This result is in line with former experiments injecting a function-blocking katanin antibody into neurons that led to reduced neurite outgrowth (Ahmad et al., 1999). However, at stages DIV12–17 when neurons have developed dendritic spines (Figs. 1–7) and are characterized by functional EPSCs (Fig. 8*A–C*), we no longer observed changes in MT growth, suggesting that, under basal conditions, functional katanin is not critical in dendrites of mature neurons.

Since both katanin subunits were detectable at glutamatergic spine synapses in colocalization with pre- and postsynaptic markers (Figs. 2, 3), we therefore asked whether katanin potentially regulates synaptic function and/or plasticity. Structural remodeling of spines follows activity-dependent triggers and requires rearrangement of its highly dynamic actin cytoskeleton (Matus, 2005; Alvarez and Sabatini, 2007; Cingolani and Goda, 2008). It is presently unclear whether and to which extent MTs participate in this process; however, our finding that functional inhibition of katanin limits spine growth following single synapse glutamate uncaging (Fig. 6) opens the possibility that dynamic MTs at least indirectly contribute to this process. Interestingly, we observed a reduction of PSD-95 following expression of GFP-p60DEID (Fig. 5), which might potentially contribute to the effect observed in the glutamate-uncaging experiment (Fig. 6), since PSD-95 is required for increasing spine size after chemical induction of LTP (Ehrlich et al., 2007).

Actin rearrangement at the base of spines is a prerequisite for microtubule polymerization into spine protrusions (Schatzle et al., 2018). Furthermore, these transient events are enhanced by synaptic activity (Merriam et al., 2011, 2013). Since actin filaments and MTs are highly interconnected at the spine base (Korobova and Svitkina, 2010), we consider the possibility that actin rearrangement and katanin-mediated MT severing cooperate in the plastic regulation of spine synapses. In general, MT severing is important to keep MTs in a dynamic state, for instance, to promote neurite branching (Baas and Qiang, 2005; Roll-Mecak and McNally, 2010; Sharp and Ross, 2012; Vemu et al., 2018). MTs within neurites are relatively long, but undergo local severing at neurite branch points. As a result, shorter MTs are thought to develop new plus ends to polymerize into newly formed branches. It has remained unclear whether a similar local severing mechanism potentially participates at the dendrite-spine interface and might contribute to the polymerization of MTs into dendrite protrusions. Since dendritic spines are oriented perpendicular to the dendrite, local severing could be critical in this process (Yu et al., 2008), because the bending capacity of MTs is limited to approximately 1.7 rad/µm (Odde et al., 1999). Therefore, the removal of tubulin subunits through the katanin (Vemu et al., 2018) could increase MT flexibility or create new seeds for MT growth into other directions. However, direct MT branching through SSNA1-mediated mechanisms (Basnet et al., 2018) could also be potentially involved. Indeed, we observed reduced EB3-labeled microtubule + TIPs in spines following inhibition of katanin function (Fig. 7). Our data provide a starting point to expand further investigations with a particular focus on actin–microtubule intersections (Nakos et al., 2022).

Further evidence that katanin is a regulator of synaptic plasticity stems from the analysis of basal synaptic transmission, as compared to the chemical induction of long-term potentiation (cLTP). Whereas katanin inhibition did not affect either EPSC amplitude or frequency under basal conditions, synaptic potentiation failed in the presence of the dominant-negative GFP-p60DEID mutant (Fig. 8). Whether this result is associated with the transient invasion of MTs into spines requires further investigation. LTP conditions induce neuronal transport of recycling endosomes (Park et al., 2004) and newly synthesized proteins (Kandel, 2001). Although MTs in spines contribute to kinesin-mediated cargo transport of postsynaptic cargo (McVicker et al., 2016), katanin inhibition might alternatively affect cargo transport along dendrites, thereby indirectly affecting postsynaptic delivery.

In summary, our data provide evidence that katanin is detectable at excitatory synapses and is critical for normal synaptic plasticity, both structural and functional LTP.

## Data availability statement

Further information and requests for resources and reagents should be directed to and will be fulfilled by the corresponding authors.
